# General Morbidity Prevalence in the Delhi Slums

**DOI:** 10.4103/0970-0218.58395

**Published:** 2009-10

**Authors:** P Marimuthu, M Hemanta Meitei, BBL Sharma

**Affiliations:** Department of Biostatistics, National Institute of Mental Health and Neuro Sciences, Bangalore, India; 1 Department of Statistics and Demography, National Institute of Health and Family Welfare, New Delhi, India

**Keywords:** Drinking water, logistic regression analysis, literacy, occupation, slum morbidity, standard of living index, toilet use

## Abstract

**Research Question:**

What is the sickness prevalence in the slums of a metropolitan city?

**Objectives:**

To estimate the morbidity prevalence with reference to a socio-economic and demographic perspective of the slum population of Delhi.

**Study Design:**

A cross-sectional study was conducted and data were collected by a two-stage random sampling method. In the first stage, slum locations were selected and in the second stage households were selected.

**Participants:**

Data were collected from 1049 households consisting of 5358 individuals' information.

**Results:**

The overall morbidity prevalence is 15.4%. It is 14.7 and 16.3% for males and females, respectively but the differences are not statistically significant. The reported higher morbidity prevalence and the illiteracy status are significantly associated. Diseases of the respiratory system appear to be very high among slum dwellers.

**Conclusion:**

From this study, it can be concluded that the number of years of staying in the slum area, presence of a separate kitchen, type of house, it being Pucca or Kuccha, types of toilet pits or open defecation are the important environmental factors for the reports of higher morbidity patterns from the slum area.

## Introduction

Slums are defined by the United Nations Organizations as “a building or group of buildings and area characterized by over crowding, deterioration in sanitary conditions, or absence of facilities and amenities, which because of these conditions or any of them endanger the health, safety or morals of its inhabitants or the community”. Local conditions, however, should be taken into account while defining the term ‘slum’.

The slums in India have been described as unsystematically developed and generally neglected. It is overcrowded, with coexistence of weak buildings, insufficient communications, and civic amenities. The existence of slums is an indication of poverty and the population dwelling in slums is termed as ‘urban poor’. According to a 2001 census(1) (India), the number of cities and towns, which accounted for the total slum population is 40 605 418, comprising 22.76% of the urban population.

It is estimated that, on an average, the slum areas of a city that contain about 20% of its population will have about 50% of all its diseases.(2) Slums are generally dirty and unclean, and have shortage of water supply, inadequate lighting and sanitation facilities. The United Nations has been more concerned with the slums of developing countries.(3) The health hazards of the urban slum dwellers are directly related to poverty and a polluted and stressful environment. They are more prone to communicable diseases and malnutrition and at the same time exposed to greater risk of accidents at work.(4)

In the last two decades, India's population has increased by 2.25%, but the urban population has increased by 3.8%.(5) An estimated 30% of the population in 12 major cities of India lives in slums and the proportion of slum dwellers and squatters have been continuously increasing. Therefore, the sanitary conditions and housing conditions of slum dwellers are deteriorating day-by-day. This calls for an urgent need for evolving a rational policy on urban resettlement.(6) Since independence, Government of India accorded high priority to rural development and rural health system. Subsequently, health facilities have expanded in the rural areas. However, the urban areas have relatively remained unattended. Urban health care services, especially in slums, have not received adequate attention. The growing urbanization unfortunately resulted in the mushrooming of slums. No comprehensive survey has been carried out either at national or state level to review the problems of slum health.(7) India has achieved a considerable reduction in the prevalence of morbidity and mortality rates and some of the communicable diseases have also been eradicated. The National Health Policy 2002 states, “The bulk of the increase is likely to take place through migration resulting in slums without any infrastructure support. Even the meager public health services, which are available, do not percolate to such unplanned habitations”.

Keeping in view, the needs of urban slum health care and data on health problems of slum population are very scanty and there is a need to study the slum morbidity patterns of major metropolitan cities in India. This will facilitate the policy makers, health administrators in the fields of health and family welfare, to plan an effective strategy for improving the health conditions of the urban slum population. Therefore, the present study attempts to estimate the morbidity prevalence with reference to socio-economic and demographic perspectives of the slum population in Delhi.

## Materials and Methods

The slum population in Delhi was considered for this study as 20% of the total population of Delhi is from slum areas.(1) A morbidity survey conducted by the National Institute of Health and Family Welfare(8) (NIHFW) reveals that the minimum prevalence (for Diarrhea) is 0.02. On the basis of this estimate and applying the sample size formula for estimating population proportion with 95% confidence interval, viz., *n*=[z^2^_1-(α/2)_ P(1-P)]/d^2^ where d=0.005 is the absolute error allowed on both sides of the estimate. Minimum of 3011.4 samples are required to arrive at the population estimate. The average family size of the slums is 5. Therefore, minimum of 603 households is to be selected from Delhi slums.

The entire city was divided into five units (East, West, North, South, and Central) and in the first stage two clusters were randomly selected from each unit. In the second stage, equal number of (around 100) households was randomly selected from the selected clusters for interview. A list obtained from the office of the Municipal Commissioner of Delhi, which had the details of slum locations and approximate number of households, was used as a sampling frame for selecting the slum locations. The households were selected after mapping and numbering the selected slum area. A well-designed interview schedule was used to interview the sample population. Tools and techniques were prepared after conducting experts' meetings with professors of Community Medicine, epidemiologists, social scientists, physiologists, and statisticians.

To reduce any recall bias, people who were suffering from any disease within 30 days was recorded.(9) All the ailments and symptoms, reported sickness, or sickness perceived by the respondent in the slum areas were recorded. Besides the social scientists and research cadre staff, post-graduate (qualified medical graduates) students of the National Institute of Health and Family Welfare (NIHFW) were involved in the data collection for this study.

### Standard of living index

Possession of the important and other household items is used as an indicator for assessing the socio-economic status of the community by different studies. This indicator is used to calculate the ‘standard of living index (SLI)’, which is similar to the method adopted by the National Family Health Survey (NFHS).(10) The advantage of having this index is that it gives single measure and it is widely recognized as proxy for socio-economic measurement. Weights for the household items are defined as follows.

**Table:** 

Item	Weight
Pressure cooker, bed, clock, and radio	One for each
Bicycle, TV, and refrigerator	Two each
Scooter and Telephone	Three each
Car	Four

Therefore, one house can get a maximum score of 20 points if it possesses all of the items mentioned above. The ‘low’ category is defined by a score of up to 5 points, a score of 6 to10 is the ‘medium’ category, and a score of 11 and above is considered as the ‘high’ category.

In each selected slum area, a locally influential person or leader was contacted and the purpose of the study was explained to him and his help and co-operation was sought for conducting the interview. The purpose of the study was also explained to the respondents and obtained their oral consent for conducting interviews. Respondents were also informed that information collected from them will be used for research purposes only and the individual information will not be shared with any other agencies or individuals.

Statistical analysis: Data was analyzed using SPSS (V 10.0) package by applying Z-test, χ^2^ test and logistic regression analysis.

## Results

### Socio-demography background

Data were collected from 1049 house-holds comprised of 5358 individuals' information. The average family size is 5.1 persons per household. The sex ratio is 789 females per 1000 males. The religious composition of the study area of the Delhi slum is: majority of them are Hindus (86.4 %) followed by Muslims (11.9 %) and Christians (1.3%). In all the age groups, the number of males outnumbers females except in the age group of ‘less than 1 year’ category. The mean age for males is 21.29 years (median = 19 years, SD = 14.98) and for females it is 20.33 years (median = 18 years, SD = 14.79) and the difference between the male and female mean age is statistically significant (*P* = 0.019). The proportions of literate are 0.738 and 0.495 for males and females respectively and the difference between these proportions was statistically significant (*P* = 0.000).

### Morbidity analysis

#### Table 1

**Table 1:** Percentage of morbidity prevalence in the households based on socio-economic variables

Characteristics	Illness reported from households	*P*
Sources of water		*P* = 0.099
Tap water	41.2	
Ground water	56.1	
Other sources	50.8	
Toilet facility		*P* = 0.047*
Flush toilet	51.6	
Public toilet	55.2	
Pit/latrine/Open defecation	56.1	
SLI		*P* = 0.024*
Low	66.7	
Medium	56.7	
High	49.3	
Fuel used for cooking		*P* = 0.042*
LPG/ Bio-gas	50.0	
Wood/Coal/Others	57.3	
Separate kitchen		*P* = 0.016*
Available	12.8	
Not available	18.2	

The morbidity prevalence of some socio-economic variables is shown in Table 1. The highest prevalence of disease is observed among the households which drink or use ground water sources. However, this recorded morbidity prevalence is statically (*P* = 0.099) not significant when considering the different sources of drinking water. From this table, we found a significant (*P* = 0.047) difference in the proportion of slum dwellers who use different types of toilets. Highest morbidity prevalence is observed among pit or latrine users. It is also to be noted that the minimum prevalence is observed among flush toilet users. Therefore, we can infer that the higher prevalence of morbidity associated among people who do not use flush toilets. This table reveals the association between the morbidity pattern and the standard of living index (SLI), High morbidity prevalence is observed among the low SLI group. The households' SLI status and morbidity pattern is negatively associated. The observed morbidity prevalence is significantly (*P* = 0.024) different over the SLI categories. LPG gas users of the households tend to have significantly less morbidity prevalence (*P* = 0.042) than those who use the other means of fuels for cooking. There is a significant difference in the reported morbidity prevalence (*P* = 0.016) between the households, which have and do not have a separate kitchen in the household. We observe from the above table that if a separate kitchen is available in the households, then a reduction of around 6% in the morbidity prevalence may be expected.

#### Table 2

**Table 2:** Distribution of specific morbidity prevalence (%) by age and sex

Age (in years)	Male	Female	All
Less than 1	22.0	16.1	18.9
1-4	21.2	17.2	19.3
5-9	9.6	11.9	10.7
10-14	8.6	8.8	8.7
15-19	10.7	9.6	10.2
20-24	11.3	12.9	12.0
25-29	11.0	18.0	14.3
30-39	16.1	22.2	18.9
40-49	26.9	24.1	25.8
50-59	27.2	33.3	29.5
60+	25.8	44.2	33.3
Total	14.7	16.3	15.4

Age-sex specific morbidity prevalence is shown in Table 1. Of the 826 sick people, 156 were infants (18.9%) suffering from some kinds of morbidity. The morbidity prevalence for male infants is higher (22.0%) than that for female infants (16.1%). The prevalence decreases with the increasing age. However, it starts increasing again from the 15 to 19 years age category to older age.

#### Table 3

**Table 3:** Distribution of morbidity prevalence (%) by education

Education levels	Male	Female	All
Cannot read and write	15.5	22.4	19.6
Can read and write	23.2	12.5	19.3
First to fifth class	10.7	12.2	10.5
Sixth to eighth class	16.9	9.3	15.8
Ninth to twelfth class	11.9	9.9	11.5
Graduates/diploma holders	14.9	-	13.5
Not applicable (< 7 years)	16.9	14.7	15.9
Total	14.7	16.3	15.4

No graduate females were recorded in the Delhi slums

Table 2 gives an account on the morbidity prevalence by the education categories. Although no clear trend emerged from the above table, we further investigated the significant differences on proportions of morbidity-prevalence between literate and illiterate categories, after merging the data (19.5% illiterate and 12.8% in the literate group were sick) and observed the significant difference (*P*=0.000) between these two proportions.

#### Table 4

**Table 4:** Logistics regression analysis

Independent variables	Exp(B)	Wald	df	*P*	95.0% C.I.for EXP(B)	
Number of years staying in slums	1.632	12.34	1	0.000	1.02	2.20
Separate kitchen in the house	1.968	3.40	1	0.045	1.32	3.01
Flesh-hut toilet (Reference category)	-	10.43	2	0.005	-	-
Public toilet	1.458	0.35	1	0.556	0.60	2.70
Pit/open defecation	2.450	10.42	1	0.001	1.11	2.98
Pucca house (reference category)	-	6.26	2	0.044	-	-
Kutccha house	1.834	5.87	1	0.015	1.01	2.4
Semi-pucca house	1.222	1.36	1	0.243	0.89	1.7
Constant	0.383	17.16	1	0.000		

Logistics regression analysis had been carried out by taking households that have reported morbidity and not reported morbidity (reference group) as dependent variable. Number of years of inhabitation in the slum areas, monthly per capita income of the household, fuel used for cooking, source of drinking water, whether a separate kitchen was available or not, flush-out toilet facility and materials used for construction of the house or hut were taken as independent variables. A step-wise backward elimination procedure was adopted for the logistics regression analysis and the final result is shown in Table 4. Number of years of staying in the slum area had an effect on reported morbidity but the effect is very little. Logistic regression analysis indicates that the houses that have a separate kitchen may have less morbidity prevalence than the houses that do not have the separate kitchen in the house. As we observed in the bi-variate analysis, it is obvious that the pit or open defecation users tend to report more morbidity than those who use the flush-toilet. The morbidity pattern of the public toilet users is also on the higher side but the odds ratio is not significant. *Wald* statistics is less and the confidence interval also indicates an unstable estimate. A similar pattern is also observed with the type of houses (*Pucca* or *Kutccha*) that exist where the slum dwellers reside. More reported morbidity patterns are observed among the people who stay in the Kutccha houses. It is also noted that higher morbidity patterns are observed among the people who reside in the semi-kutccha houses, but the calculated odds ratio is not significant and the confidence interval also indicates the unstable estimate of odds ratio.

## Discussion

A study on adolescents of Delhi slums(11) reveals that out of 90 subjects, 34 had skin ailments, one had an enlarged thyroid gland, six had enlarged lymphoids, 16 had eye ailments, 21 had ENT problems, and 13 had positive findings concerned with genitor-urinary system but not affected by STD. In spite of the well-known fact that concentration of health resources in cities, proximity of hospitals, and other medical facilities are better than the rural areas, the standard of health care services falls far below minimum level for those who live in urban slums. Another study(12) on health care delivery in an urban slum of Delhi revealed that negligence of preventive services by dispensaries of Delhi administration, inadequate maternal and child health services are some of the factors for improper health delivery system in slums.

A study on diarrheal diseases of Delhi slums (by Bhatnagar and Dosajs, 1986) revealed that the incidence of diarrhea averaged 8 episodes per child per year. And in 1988 Bhatnagar(13) and his team documented that maximum sickness was registered in slums having the poorest sanitary conditions. Important correlates of morbidity were identified as low educational level, poor hygienic status of the family, poor environmental sanitation and low per-capita income. SC Gulati(14) and his team had conducted a survey in the Delhi slums, called Reproductive Health in Delhi Slums. The main objective of this study is reflected in the title itself. They have covered a sample of 500 households from five different slum locations. They concluded that benefits of Reproductive and Child Health programs are yet to reach the urban poor.

In the Ludhiana study, about 55% of children of 6-42 months who were malnourished had history of recent diarrheal disease.(15) Udani Pekha H(16) had conducted a study in the urban slums which were under an ICDS block in Bombay, to find out the morbidity pattern and nutritional status of pre-school children of these slums. Twelve out of 25 slums were randomly selected for the study. These children were carefully examined by four teams of pediatricians and reported the following ailments, upper respiratory tract infection, scabies and physical and mental disabilities were some of the leading diseases. Indke(17) *et al*, investigated the poliomyelitis in India's largest slum in Mumbai. They reported that among children less than 6 years, male-female impairment ratio was 1 : 5. Authors viewed that this difference could be due to high-case fatality in female children. Kushwaha KP(18) *et al*, studied the superstitious therapy during illnesses of pre-school children. Superstitions were more common in the lower socio-economic group. Out of 2278 episodes of diseases in which superstitious therapy was used, 0.1%belonged to social class I, 26.1% to class II, 28.1% to class III, 5.9 % to class IV, and 1.2% to class V.

In conclusion, we emphasize that the lifestyle of the urban poor that is associated with ill health and the environment in which they live are positively correlated with morbidity prevalence. We have observed herein that the number of years of staying in slums is positively associated with morbidity prevalence. Of course, the size of the houses is also an important factor, which is directly associated with prevalence of morbidity in the households viz., the availability of a separate kitchen has an inverse effect with reported morbidities. Another factor, which is related with prevalence of higher morbidity, is the type of house in which they live. People who stay in kuttcha or semi-kutccha houses have increased morbidity pattern than those who stay in the pucca houses. So, we can infer from this study that the number of years of staying in the slums, size of the households, and the material used for constructing the households are primary environmental factors associated with higher morbidity. A major public health problem in the slum areas is availability of proper toilet facilities. Our study clearly indicates that the people who are not using flush toilets are reported with higher morbidity. Although many Non-Governmental Organizations work to provide proper toilet facilities in the slum areas, there are new slum areas rising now and then in the city that pose a greater challenge to the government and to voluntary organizations.
